# Catalytic Activity of LaFe_0.4_Ni_0.6_O_3_/CeO_2_ Composites in CO and CH_4_ Oxidation Depending on Their Preparation Conditions

**DOI:** 10.3390/ma16031142

**Published:** 2023-01-29

**Authors:** Lyubov Isupova, Evgeny Gerasimov, Igor Prosvirin, Vladimir Rogov

**Affiliations:** Boreskov Institute of Catalysis SB RAS, 630090 Novosibirsk, Russia

**Keywords:** LaFe_0.4_Ni_0.6_O_3_/CeO_2_ composites, preparation, methane, CO oxidation

## Abstract

LaFe_0.4_Ni_0.6_O_3_/CeO_2_ (1:1) two-phase composite materials were prepared by mechanochemical (MC) and Pechini routes. The catalytic properties of the composites in methane and CO oxidation reactions strongly depend on their preparation conditions. In low-temperature (<600 °C) catalytic CO oxidation the composites demonstrate a higher activity compared with LaFe_0.4_Ni_0.6_O_3_ perovskite. The highest activity was observed for the composite prepared by mechanical treatment of perovskite and fluorite precursors. There is a correlation between activity and the content of weakly bound surface oxygen species. Catalytic activity in high-temperature (>750 °C) catalytic methane oxidation correlates with the reducibility of samples. The highest activity was observed for the composite prepared by the one-pot Pechini route with higher reducibility of the sample up to 600 °C.

## 1. Introduction

Progress in high-temperature processes such as membrane, catalytic, SOFC, etc., depends on the development of new active and stable functional materials with high conductivity, bulk oxygen mobility, and catalytic activity. It is very difficult to find a monophasic material satisfying all these demands. One of the ways to solve the problem is the preparation of composite materials consisting of two phases with different properties. Among them, perovskite–fluorite composites are very promising for high-temperature applications due to the possibility to form coherent interphase boundaries and combine properties of constituent components (high bulk oxygen mobility of fluorites with a high rate of oxygen heteroexchange as well as electron conductivity and catalytic activity of perovskites) [1,2,3,4,5]. In addition, synergistic effects were observed in such two-phase systems. Thus, the application of CeO_2_-based supports in the synthesis of supported perovskite catalysts enhances the activity in oxidation reactions, which is attributed to the non-additive effect upon the interaction of these phases [6,7,8]. A similar result was noted when studying the La_0.8_Sr_0.2_MnO_3_/MeO_x_ (Me = Sr, La, Ba, Ce) composites obtained by mechanochemical treatment of La_0.8_Sr_0.2_MnO_3_ and MeO_x_ oxides, in which the non-additive effect was observed only in the case of Me = Ce and became more pronounced with increasing calcination temperature of the composite [9]. The authors related this to the unique properties of the CeO_2_–high content of oxygen and its ability (in comparison with other oxides) to readily exchange with the environment, which increases when CeO_2_ is modified with other cations.

In a recent review on composites in methane oxidation, the clear evidence of mixed oxide cerium-containing catalysts in achieving highly active catalysts in complete methane combustion was demonstrated as well as that such catalysts may be valid alternatives to the more expensive and sensible noble metal catalysts. Ce_1−x_Fe_x_O_2−x_ (x = 0.3−0.4) and 20% NiO/CeO_2_ composites were shown to be among the most active. Their activity in methane oxidation was comparable with the activity of the industrial catalyst Pd/Al_2_O_3_ [10].

The effect of phase ratio in a composite on its properties was studied in some works [4,11]. The authors of [11] investigated (0.1 or 0.8) LaFeO_3_/CeO_2_ composites, which were obtained by mechanical activation of the calcined oxides, in methane oxidation and nitrous oxide decomposition reactions. The non-additive effect was shown to manifest itself only after thermal treatment of the composites. In the oxidation of methane, the composites, despite a smaller content of LaFeO_3_, compared well in activity with LaFeO_3_, whereas in the decomposition of nitrous oxide their activity strongly exceeded that of LaFeO_3_. The study did not reveal an essential difference in activity (reaction rate) of the composites with different (0.1 or 0.8) CeO_2_ content in the tested reactions. At the same time, a wider variation of the phases ratio in the La_0.65_Sr_0.35_MnO_3_/CeO_2_ composites demonstrated that the maximum oxygen exchange rate (which is important for the red-ox processes where reoxidation of the active site serves as the limiting step) was achieved exactly at the 1:1 ratio [4].

One of the promising composites for application in different high-temperature red-ox processes—catalytic, membrane, SOFC, and others—is the LaFe_0.4_Ni_0.6_O_3_/CeO_2_ composite due to the high catalytic activity, mixed conductivity, and high-temperature stability of LaFe_0.4_Ni_0.6_O_3_ [12,13,14,15]. For example, LaFe_1-x_Ni_x_O_3_ (x = 0.2–0.8) catalysts showed high activity and resistance to coking in carbon dioxide and steam reforming of methane, in distinction to LaFeO_3_ and LaNiO_3_ [12,13]. In [14,15], high conductivity of LaFe_0.4_Ni_0.6_O_3_ was observed, which made this material very promising for use in medium-temperature solid oxide fuel cells (SOFC). Investigation on the compatibility of LaFe_0.4_Ni_0.6_O_3_ with CeO_2_ or ZrO_2_, which was performed in [16,17], revealed a higher stability of LaFe_0.4_Ni_0.6_O_3_/CeO_2_ composites. The acquired data on the conductivity of the composites in dependence on the perovskite content suggest that by increasing the perovskite content above 50% the main contribution to conductivity is made by the electronic conductivity, whereas at a perovskite content below 50%, the main contribution is made by the ionic one. At the 1:1 ratio of components, the composite simultaneously possesses high electronic and ionic conductivity, which is essential for its application as the SOFC anodes and cathodes. A similar dependence of conductivity on the perovskite content was obtained in [18] for the composites prepared using mischmetals (nonseparated mixtures of lanthanides with different contents of lanthanum).

The data available in the literature allow assuming that the LaFe_0.4_Ni_0.6_O_3_/CeO_2_ composite with the phase ratio 1:1 will be highly active and stable in high-temperature methane and CO oxidation reactions, which is interesting for its application both as the deep oxidation catalyst and the material for direct SOFC.

The LaFe_0.4_Ni_0.6_O_3_/CeO_2_ composite can be prepared in different ways in one or two stages before calcination. The perovskite and fluorite oxides previously prepared via different routes (the first stage) are usually combined in the second stage, and after that, the obtained mixture of oxides is calcined to form a composite material (the preparation in two stages) [4,11,19]. A one-stage (or one-pot) preparation route means that all raw materials are combined at the first stage with the formation of a precursor, which forms a composite material after calcination. New possibilities for composite preparation have emerged due to the development of the mechanochemical (MC) method, which may be applied at different stages: for preparation of perovskites (the first stage) [20,21,22], for combination of perovskite and fluorite phases (the second stage) [11,23], and for one-pot preparation of composite materials [24]. The MC method makes it possible to also prepare materials without wet stages and waste water [20,21,22].

The properties of composite materials are determined not only by the properties of constituent components and their quantitative ratio, but also by the preparation method that influences the particle size of constituent phases, homogeneity of the particles (phases) distribution and their mutual modification, as well as the length of interphase boundaries that affect the properties of the composites [10,24,25,26].

It is reputed that the best methods for low-temperature LaFe_0.4_Ni_0.6_O_3_ preparation are Pechini, sol–gel, and co-precipitation methods [12,16,27,28]. The ceramic method, being the simplest one, requires a long high-temperature (>1200 °C, >100 h) calcination [14]. The mechanical treatment of raw materials in high-power planetary ball mills, due to their disintegration, homogenization, and activation, also provides the possibility of low-temperature preparation of a LaFe_0.4_Ni_0.6_O_3_ perovskite and LaFe_0.4_Ni_0.6_O_3_/CeO_2_ composite.

The main goal of this paper is the preparation of LaFe_0.4_Ni_0.6_O_3_ perovskite and LaFe_0.4_Ni_0.6_O_3_/CeO_2_ (1:1) composites using mechanical and Pechini routes and investigation of their physical and chemical properties including catalytic activity in CO and CH_4_ oxidation processes.

## 2. Materials and Methods

### 2.1. Materials Synthesis

For MC preparation of LaFe_0.4_Ni_0.6_O_3_, a mixture of lanthanum, iron, and nickel oxides in the required stoichiometry was treated in a high-power planetary ball mill VRR835V/4B for 3 min at a powder to ball ratio of 1:5. The activated powder mixture was then calcined at 900 °C for 6–100 h with several intermediate millings.

A LaFe_0.4_Ni_0.6_O_3_ (sample 1), CeO_2_ (sample 2), and a LaFe_0.4_Ni_0.6_O_3_/CeO_2_ composite with a La:Ce ratio of 1:1 (sample 3) were prepared via the Pechini method [27,28] using nitrate salts that were applied in the required proportion. After drying of the amorphous precursors (1–3), the obtained powders were mechanically treated in the high-power planetary ball mill for 3 min and then calcined at 900 °C for 8 h in air. A LaFe_0.4_Ni_0.6_O_3_/CeO_2_ composite (sample 4) was also prepared using the calcined LaFe_0.4_Ni_0.6_O_3_ (sample 1) and CeO_2_ (sample 2) oxides that were generated with the La:Ce ratio 1:1. After their joint mechanical treatment in the high-power planetary ball mill for 3 min, the powder was additionally calcined at 900 °C for 8 h. The LaFe_0.4_Ni_0.6_O_3_/CeO_2_ composite (sample 5) was also prepared using amorphous precursors of samples 1 and 2 (before their calcination). Precursors 1 and 2 taken with the La:Ce ratio of 1:1 after their joint mechanical treatment in the high-power planetary ball mill during 3 min were also calcined at 900 °C for 8 h.

In summary, a pure perovskite LaFe_0.4_Ni_0.6_O_3_ (sample 1), a fluorite CeO_2_ (sample 2), and three LaFe_0.4_Ni_0.6_O_3_/CeO_2_ composites with a La:Ce ratio of 1:1 were prepared. Composite 3 was prepared by the Pechini route in one stage, whereas composites 4 and 5 were prepared in two stages using different precursors. Details of the samples preparation are indicated in Table 1.

### 2.2. Materials Characterization

X-ray diffraction (XRD) patterns were obtained using an X’TRA (Thermo ARL, Switzerland) diffractometer with Cu*Kα* monochromatic radiation. Each sample was scanned in the 2θ range from 10 to 70° with a 2θ step of 0.05°. The X-ray crystallite sizes (CS) and phase compositions were calculated in the X’Pert HighScore Plus (PANalytical B.V., Almelo, The Netherlands) software. The calculation and refinement of lattice parameters were performed in the Polycrystal software package [29] (BIC SB RAN, Novosibirsk, Russia) software by the method of least squares.

Transmission electron microscopy (TEM) micrographs were obtained with a JEM-2010 instrument with a lattice resolution of 1.4 Å and an acceleration voltage of 200 kV (JEOL, Japan).

EDX mappings were conducted using a ThemisZ transmission electron microscope (Thermo Fisher Scientific, USA) operated at an accelerating voltage of 200 kV. The microscope was equipped with a SuperX spectrometer (Thermo Fisher Scientific, USA) for EDX mapping measurements. For electron microscopy studies, samples were deposited on perforated carbon substrates attached to copper grids using an ultrasonic dispersant with ethanol.

Surface composition of the samples was investigated by X-ray photoelectron spectroscopy (XPS) using a SPECS spectrometer with a PHOIBOS-150-MCD-9 hemispherical energy analyzer (Al K_α_ irradiation, hv = 1486.74 eV, 200 W). The samples were secured with a double-sided conducting copper scotch tape. The binding energy (BE) scale was preliminarily calibrated against positions of the peaks of Au4f_7/2_ (BE = 84.0 eV) and Cu2p_3/2_ (BE = 932.67 eV) core levels. The binding energy of peaks was calibrated against the position of the C1s peak (BE = 284.8 eV) corresponding to the surface hydrocarbon-like deposits (C-C and C-H bonds). The ratio of surface atomic concentrations of the elements was calculated from the integral intensities of photoelectron peaks corrected by the corresponding atomic sensitivity factors based on Scofield photoionization cross-sections [30]. Analysis of the data obtained by XPS was carried out by the software XPS Peak 4.1. [31].

The data on the samples’ thermoprogrammed reduction with hydrogen (H_2_-TPR data) were obtained using a flow reactor equipped with a thermal conductivity detector. Samples of 200 mg with a particle size of 0.25–0.5 mm were used for H_2_-TPR. Before the reduction, the samples were treated in an oxygen flow at 500 °C for 0.5 h and then cooled to room temperature. The samples were heated (10 °C/min) in a gas (10% H_2_ in Ar) flow (40 cm^3^ min^−1^) up to 900 °C.

The specific surface area of the samples was measured using Ar desorption at 200 °C.

### 2.3. Catalytic Activity

The catalytic activity was characterized in terms of conversion (X) and rates of CO or CH_4_ oxidation (*w* = 2.69 ×10^19^ *kC*_0,_ molecules ·m^–2^ s^–1^, where *k* is the rate constant, and C_0_ is the initial concentration of methane or CO, %).

Catalytic activity in CO oxidation was determined using a circulating flow reactor (a circulation rate of 900–1000 l·h^−1^) in the range of 200–600 °C. The sample weight was 1 g, the reaction gas (1% CO + 1% O_2_ + 98% N_2_) flow rate was 10 l·h^−1^, and the contact time (τ) was 0.2 s. The rate constant *k* was calculated under the assumption of perfect mixing mode of the reaction using the formula

*k* = *X*_CO_·(1−*X*_CO_)^−1^·g^−1^·S_sp_^−1^·τ^−1^ (1)

where *X*_CO_ is the CO conversion; g—the catalyst weight, g; S_sp_—the specific surface area, m^2^·g^−1^; and τ—the contact time, s.

Catalytic activity in methane oxidation in the range of 600–900 °C was determined in the flow reactor with a gas mixture (1% CH_4_ + 9%O_2_ + 90%He) at a flow rate of 60 l·h^−1^ and a contact time (τ) of 0.006 s. Particles of 0.5–0.25 mm size were used for all the experiments. The products of methane oxidation were only carbon dioxide and water. The rate constant *k* was calculated under the assumption of the plug-flow mode using the formula

*k =*−ln(1 − X_CH_4__)·τ^−1^·S_sp_^−1^·g^−1^ (2)

where X_CH_4__ is the methane conversion; τ—the contact time, s; *g*—the sample weight, g; and *S*_sp_—the specific surface area of the sample, m^2^·g^−1^.

## 3. Results and Discussion

### 3.1. Phase Composition

According to the XRD data, doped LaFeO_3_, LaNiO_3_, La_2_NiO_4_, and simple oxides (La_2_O_3_, Fe_2_O_3_, NiO) were detected in the mixture of the MC-treated initial raw oxides. The subsequent calcinations at 900 °C for 6–100 h led to an increase in the amounts of both (based on LaFeO_3_ and on LaNiO_3_) doped perovskite phases, but the monophase LaFe_0.4_Ni_0.6_O_3_ perovskite was not detected even after calcination during 100 h, probably due to very mild MC treatment conditions. Therefore, for our subsequent experiments on composites preparation, the monophase LaFe_0.4_Ni_0.6_O_3_ (perovskite) and CeO_2_ (fluorite) oxides and their precursors prepared via the Pechini method were used, while MC treatment was applied only for their combination. For comparison, the composite prepared via the one-pot Pechini route (sample 3) was also studied.

**Figure 1:**
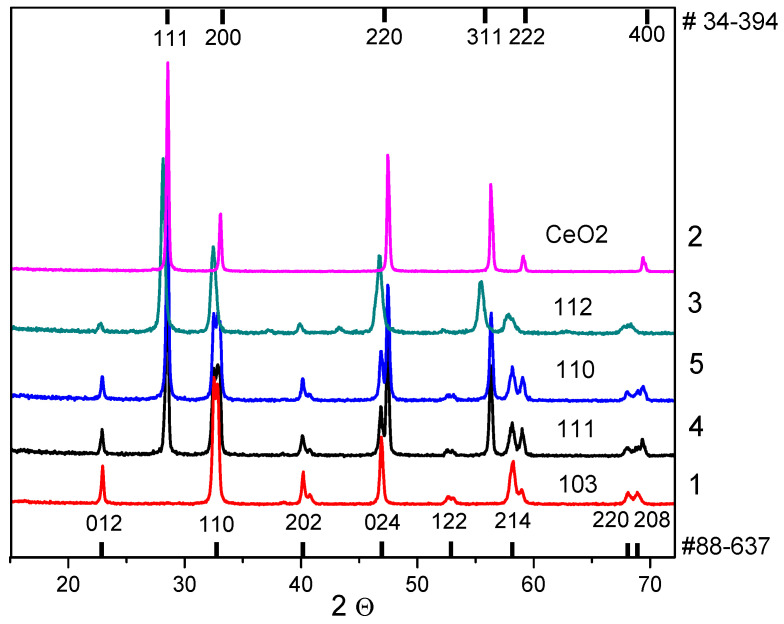
XRD patterns for LaFe_0.4_Ni_0.6_O_3_/CeO_2_ samples. Curve numbers (1–5) correspond to the sample numbers (Table 1).

The XRD data for the as-prepared samples (Figure 1) indicate the presence of only the perovskite phase (sample 1) according to PDF card #88-637, only the fluorite phase (sample 2) according to PDF card #34-394, and both (perovskite and fluorite) phases in the composites (samples 3–5). It is worth noting that cerium oxide is observed in trace amounts in sample 3, so the cell parameters for this phase were not calculated. The perovskite phase in samples 1, 4, and 5 was determined in the rhombohedral modification (PDF card #88-637) with a small difference in cell parameters, while in sample 3 it was determined in the orthorhombic modification (PDF card #88-638) with a noticeable difference in cell parameters (Table 2), which may be due to the weak (samples 4 and 5) or strong (sample 3) modification of the perovskite structure with Ce cations in the as-prepared composites. A similar modification of the perovskite phase was observed in the work of Ren et al. [32] for chemical compositions La_0.6_Ce_0.4_FeO_3_ and La_0.8_Ce_0.2_FeO_3_. This effect can be explained by a significant difference in the cationic radii of lanthanum and cerium. The partial entry of cerium cations into the perovskite structure was also confirmed by the absence of visible reflexes on diffractograms in sample 3 as previously mentioned above.

The X-ray crystallite sizes for both phases (perovskite and fluorite) detected in the composites depend on the samples preparation conditions. For both phases, the sizes are nearly 50 nm in the monophase samples (1 and 2), nearly 40 nm in the samples prepared via the MC combination of oxides or their precursors (samples 4 and 5, respectively), and nearly 25 nm in the one-pot prepared composite (sample 3). The observed differences in the perovskite and fluorite cell parameters as well as in the X-ray crystallite sizes detected for the one-pot prepared composite (sample 3) compared with the other two composites (samples 4 and 5) or monophase samples (samples 1 and 2) revealed a strong bulk chemical modification of both the perovskite and fluorite phases in the one-pot prepared composite (sample 3). The surface mutual modification of the perovskite and fluorite phases can be proposed for composites 4 and 5 mainly because of the smaller changes in the oxides’ cell parameters according to XRD (Table 2). The data on the samples’ specific surface area values are listed in Table 2.

### 3.2. Microstructure, Bulk, and Surface Particles Composition

**Figure 2:**
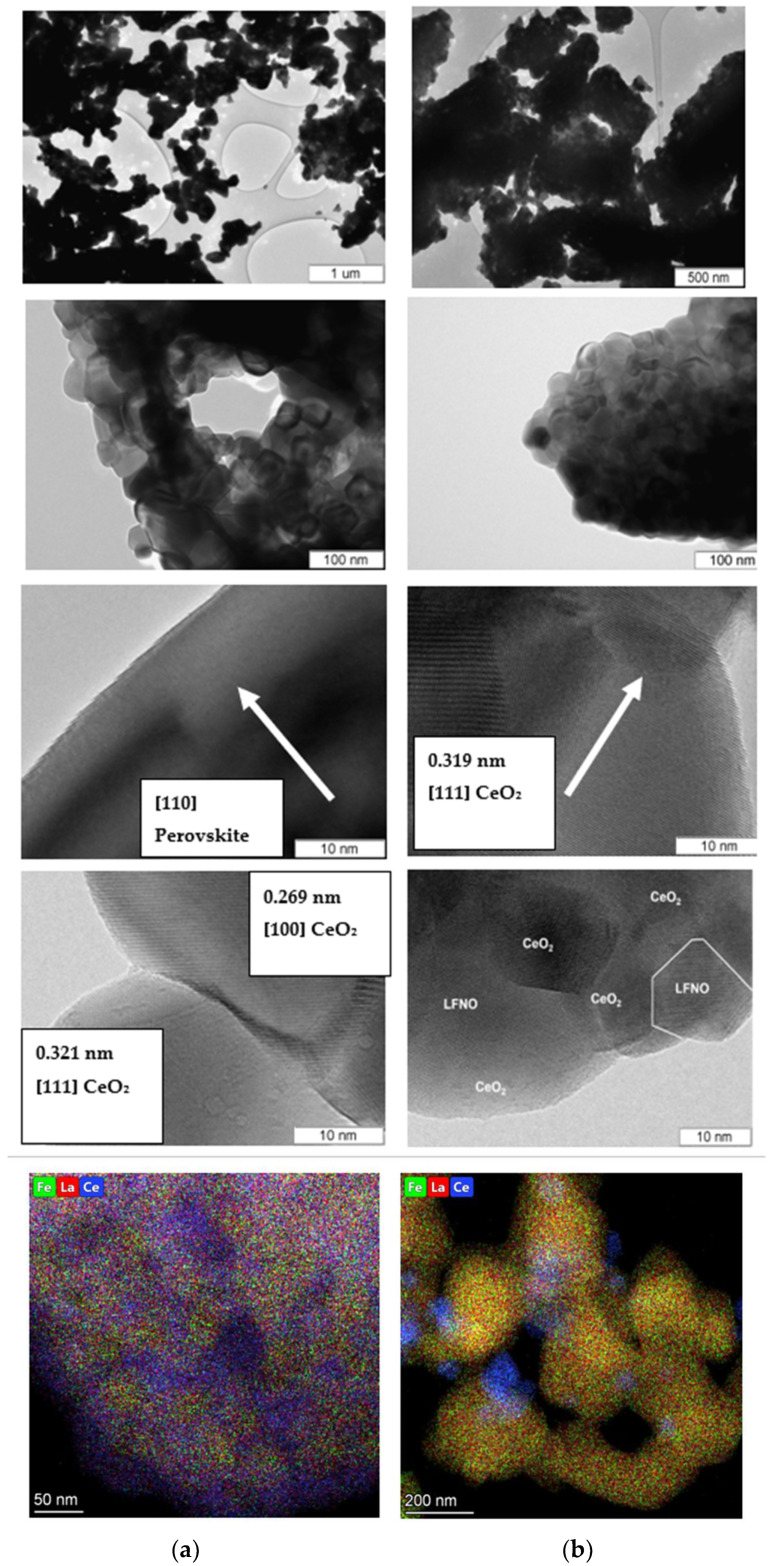
TEM data for LaFe_0.4_Ni_0.6_O_3_/CeO_2_ composites 3 (**b**) and 5 (**a**) at different magnifications and EDX mapping for 3 (**b**) and 5 (**a**) samples.

TEM and EDX mapping (Figure 2) data revealed the generally separated perovskite (one type) and fluorite (another type) aggregated particles (both about 1 μm in size) in composites 4 and 5 and only the mixed (perovskite + fluorite) aggregated particles with nearly the same size (~ 1 μm) in composite 3. All the aggregates were composed of 20–50 nm crystallites, which is consistent with the crystallite size range estimated by XRD (Table 2).

According to the EDX data obtained from different local areas in different aggregated particles, the aggregates in sample 1 are fairly uniform in composition. The enrichment of the particle surface with La may be proposed for this sample due to its higher content. Two types of local areas that strongly differ in composition (enriched with perovskite or fluorite cations) were distinguished in samples 4 and 5 in different aggregates consisting mainly of perovskite or fluorite phases, respectively. One type of aggregate particles and areas composition was revealed in sample 3, which is fairly uniform in composition in different aggregates. Due to a higher Ce content in the analyzed areas, the enrichment of the particles’ surface with Ce may be proposed for sample 3 since the signal from cerium on the surface of agglomerates is quite clearly visible on the EDX mapping (Figure 2a).

Hence, according to the TEM + EDX data, two types of aggregates in samples 4 and 5, which differ in chemical composition, and only one type in sample 3 were revealed for the prepared two-phase composites (samples 3–5). It is clear that the homogeneity of the LaFe_0.4_Ni_0.6_O_3_ and CeO_2_ phases’ mutual distribution and their mutual modification are much higher in sample 3 compared with samples 4 and 5.

**Figure 3:**
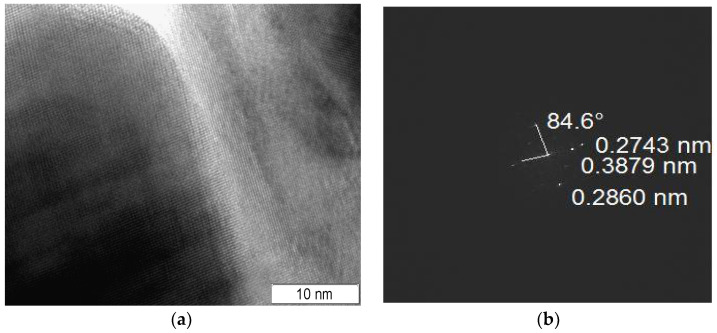
Interphase boundary (**a**) and its Fourier transform image (**b**). Sample 5.

Due to the difference between X-ray crystallite size and aggregate size, the formation of interblock and interphase boundaries is very possible in the as-prepared samples. One may propose a higher density of interphase boundaries in sample 3 due to the formation of the mixed aggregates and smaller X-ray perovskite and fluorite crystallite sizes. However, the formation of interphase boundaries was detected in other composites (samples 4 and 5), too. In Figure 3, there is an area between the perovskite and fluorite crystallites in which the Fourier transform image confirms the formation of interphase boundaries in composite 5 because the detected interplanar spacings correspond to perovskite (0.2860, 0.3879 nm) and fluorite (0.2743 nm) structures.

**Figure 4:**
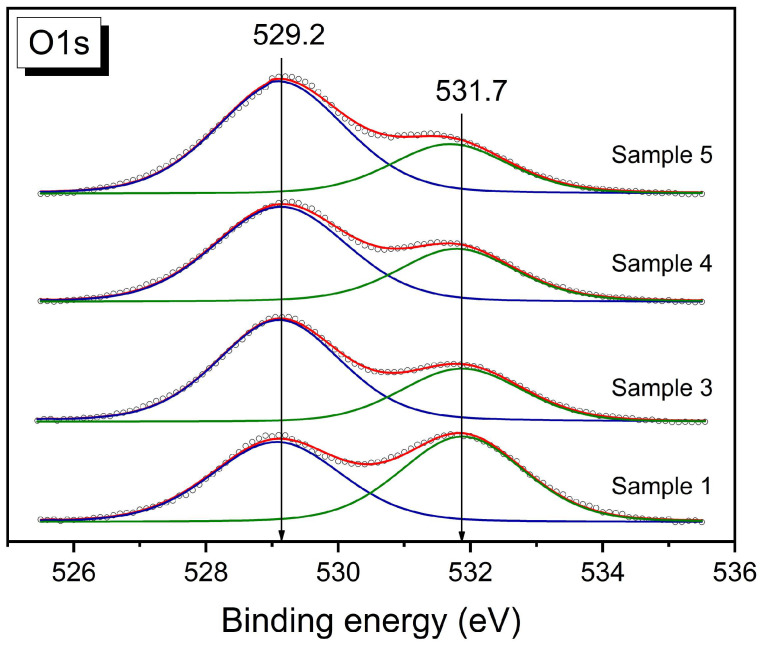
O1s XPS spectra for LaFe_0.4_Ni_0.6_O_3_/CeO_2_ samples.

It should be noted that, compared with X-ray diffraction, X-ray photoelectron spectroscopy is a surface-sensitive method and the depth of analysis is approximately 6–10 nm, depending on the kinetic energy of the photoelectrons [33]. According to the XPS data, the surface of the samples is enriched with oxygen in the perovskite and fluorite structures with BE (O1s) = 529.2 ± 0.1 eV (Figure 4, blue lines) and, very probably, in the surface carbonates because there is oxygen with BE (O1s) = 531.7 ± 0.2 eV (Figure 4, green lines) and carbon (BE (C1s) = 288.9 ± 0.2 eV) ions in the carbonates [30,34]. Samples 3–5 may contain a lower quantity of surface carbonates because of a higher O_(529)_/O_(531)_ ratio. The surface of all the composites is also enriched with “perovskite” (La, Fe, and Ni) ions compared with the monophase perovskite sample (Table 3). The samples differ in their La/Ce ratio as well as the (Fe + Ni)/(La + Ce) and (Fe + Ni)/La ratios (Table 3). The highest content of “perovskite” ions was revealed for sample 4 (La/Ce = 1.36 and (Fe + Ni)/La = 0.61). Although for sample 5 the La/Ce ratio is lower (La/Ce = 0.86), the (Fe + Ni)/La ratio is higher (0.64) than that for sample 4 (0.61). The lowest (Fe + Ni)/La ratio (0.48) at La/Ce = 1.06 was revealed for the composite prepared via the one-pot Pechini method (sample 3). Hence, according to the XPS data, surface enrichment with “perovskite” ions (La + Fe + Ni) in the composites was revealed. Taking into account the highest surface content of the “perovskite” ions in composites 4 and 5 compared with composite 3 and with its possible content (at Perovskite: Fluorite = 1: 1 ratio and near the same particles and X-ray sizes), a significant modification of the surface of the fluorite particles in samples 4 and 5 with La, Fe, and Ni ions in the perovskite and/or simple oxide forms may be proposed for the samples; this is consistent with the data obtained and discussed in detail in [11], while the bulk modification with “perovskite” ions may mainly be proposed for sample 3, which agrees with the X-ray data.

Therefore, according to the XPS data, a higher surface content of “perovskite” ions was revealed for all the composites (samples 3–5) compared with pure perovskite (sample 1). The composites may also be depleted with carbonates. The main difference between the prepared composites is a higher surface content of 3d ions in the MC samples (samples 4 and 5) compared with the Pechini sample (sample 3).

### 3.3. H_2_-TPR Data

**Figure 5:**
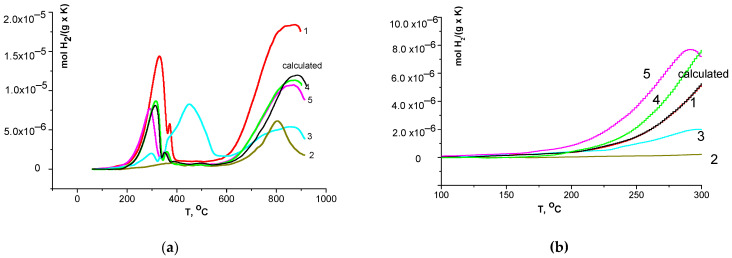
H_2_-TPR data (**a**) and low temperature consumption (**b**) for samples. Curves designations correspond to sample numbers (Table 1). Calculated (black line)—he calculated data for the mixture LaFe_0.4_Ni_0.6_O_3_ + CeO_2_ in a 1:1 ratio.

According to H_2_-TPR (Figure 5a), there are two main peaks of hydrogen consumption for LaFe_0.4_Ni_0.6_O_3_ perovskite (sample 1) and LaFe_0.4_Ni_0.6_O_3_/CeO_2_ composites (samples 3–5), while only one high-temperature peak was revealed for CeO_2_ (sample 2). No strong difference in the total (up to 900 °C) hydrogen consumption for composites 4 and 5 was detected (Table 4). Their total consumption (~3.15·10^−3^ mol H_2_·g^−1^) is lower than that calculated for a mixture of perovskite (sample 1) and fluorite (sample 2) oxides in the 1:1 ratio (3.66·10^−3^ mol H_2_·g^−1^) and points to the bulk modification of both phases in the as-prepared composites that led to oxygen vacancy formation in both oxides. The H_2_-TPR data for sample 3 strongly differ from that of other composites. A lower total (up to 900 °C) hydrogen consumption (2.81·10^−3^ mol H_2_·g^−1^) detected for sample 3 compared with samples 4 and 5 may be due to a stronger mutual bulk chemical modification of constituent phases in the one-pot prepared composite, resulting in the higher oxygen vacancy formation in both phases.

For composites 4 and 5, H_2_ consumption in the first reduction peaks is also lower (0.61–0.64·10^−3^ mol H_2_·g^−1^) than the value calculated for the mixture of oxides (0.7·10^−3^ mol H_2_·g^−1^), while for composite 3 the consumption in the first peak is much higher (1.38·10^−3^ mol H_2_·g^−1^) compared with the calculated value. Therewith, consumption in the first reduction peaks for samples 4 and 5 is lower compared with sample 1, and the temperature maximum of the main reduction peak for sample 3 is much higher compared with the other composites (~200 °C). The detected changes in the temperature maximum of the first reduction peaks for samples 3, 4, and 5 (Figure 5a) may also be due to the longevity of mutual modification of the phases for sample 3.

As for the hydrogen consumption at lower temperatures (up to 300 °C), which may be due to the removal of the most weakly bound surface oxygen species (that is important for low-temperature oxidative processes [35]), it decreases in the series 5 > 4 > 3 (Figure 5b), which correlates with specific surface areas of the samples (m^2^·g^−1^, 7.3 > 5.2 > 2). The H_2_ consumption calculated for the mixture at temperatures up to 300 °C is lower than that for samples 4 and 5 but higher than for sample 3 (Figure 5b). The higher low-temperature consumption for composites 4 and 5 compared with the calculated value at near the same values of specific surface areas may be due to the formation of surface oxygen vacancies in the modified perovskite and fluorite phases with the weakly bound oxygen species incorporated into vacancies. The smaller amount of the weakly bound oxygen species in sample 3 may be due to its lower specific surface area.

### 3.4. Catalytic Activity in CO and CH_4_ Oxidation

**Figure 6:**
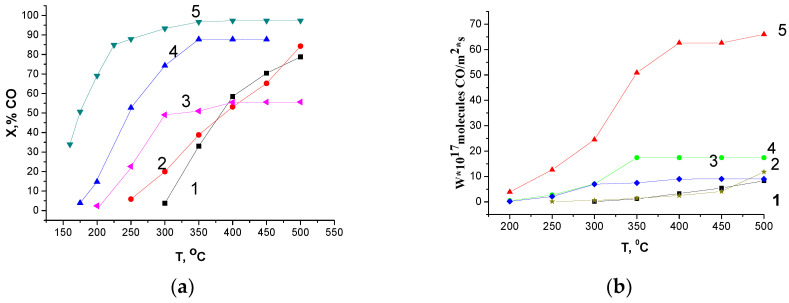
Conversion of CO (**a**) and rate of CO oxidation (**b**) for the as-prepared samples. The gas mixture (1% CO + 1% O_2_ + 98% N_2_) flow rate is 10 L/h; circulation 900–1000 L/h, and contact time (τ) is 0.2 s. Curves designations correspond to sample numbers (Table 1).

In spite of the lower LaFe_0.4_Ni_0.6_O_3_ content in the composites, their catalytic activity (conversion) in CO oxidation is much higher than the monophase activity of the LaFe_0.4_Ni_0.6_O_3_ and CeO_2_ samples (Figure 6a).

The conversion of CO for composites 5 and 4 was higher than that for the composite prepared via the one-pot Pechini route (sample 3). Actually, there is a correlation between the low-temperature hydrogen consumption (up to 300 °C) and the catalytic activity of the samples in CO oxidation. Hence, the difference in activity may be due to the difference in their specific surface areas but catalytic activities normalized to m^2^ (Figure 6b) were in the same (5 > 4 > 3 > 1 = 2) order. Therefore, the clear non-additive effect in CO oxidation was revealed for all the composites due to the modification of CeO_2_ with La and 3d ions and the formation of point defects in the CeO_2_ subsurface, which led to an increase in the content of weakly bound surface oxygen species adsorbed on vacancies and the low-temperature activity of the prepared composites. It should be noted that the activity (at 300 °C) of composite 5, which was prepared by the mechanochemical method (W = 2.5·10^18^ CO molecules·m^−2^ s^−1^), exceeds virtually 5-fold the activity (measured at the same conditions) of LaMnO_3_, which is one of the most active perovskites in the oxidation reactions (W = 0.45·10^18^ CO molecules·m^−2^·s^−1^ according to [35]). However, the low-temperature activity in CO oxidation is lower than the activity of the most active composite Cu_0.1_[Ce(La)]_0.9_O_x_, which at 200 °C and a contact time of 0.09 s in a reaction mixture of 2% CO + 16% O_2_ demonstrated 100% conversion, very probably due to the much higher specific surface area value [36].

**Figure 7:**
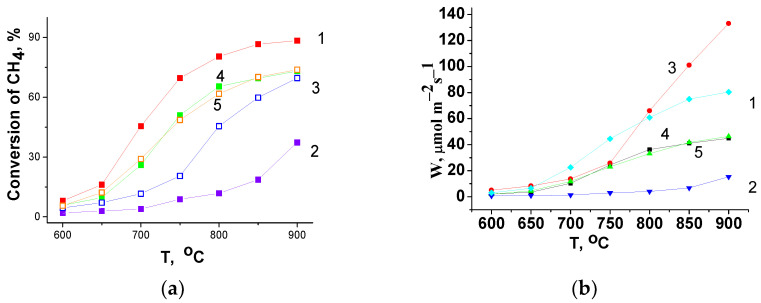
Conversion of methane (**a**) and CH_4_ oxidation rates (**b**) for the as-prepared samples. The gas mixture (1% CH_4_ + 9%O_2_ + 90%He) flow rate is 60 L/h and contact time (τ) is 0.006 s. Curves designations correspond to sample numbers (Table 1).

In high-temperature (600–900 °C) methane oxidation, LaFe_0.4_Ni_0.6_O_3_/CeO_2_ composites (samples 3–5) demonstrate a lower conversion than LaFe_0.4_Ni_0.6_O_3_ (sample 1), while the CeO_2_ (sample 2) shows the lowest value (Figure 7a). The one-pot prepared composite (sample 3) demonstrates the lowest conversion, but with an increase in the testing temperature the difference in conversions between composites decreases and at 900 °C all the composites show nearly the same conversion. The reaction rate of methane oxidation at 900 °C obtained for the composite strongly exceeds the value for LaMnO_3_, which is equal to 8 µmol·m^−2^·s^−1^, and even the oxidation rate for a more active La_0.5_Sr_0.5_MnO_3_ (100 µmol·m^−2^·s^−1^) according to [37]. Due to very different testing conditions in methane oxidation, it is difficult to compare the high-temperature activity of the prepared composites with the low-temperature activity of the most active ones according to [10,36]. 

Actually, there is a correlation between the hydrogen consumption in the first peak (up to 400 °C) and the catalytic activity (Figure 7a) of the samples in CH_4_ oxidation. The catalytic activity of the samples normalized to m^2^ (Figure 7b) also revealed a lower activity of the composites compared with LaFe_0.4_Ni_0.6_O_3_ perovskite at temperatures up to 750 °C. At higher temperatures (T > 750 °C), the normalized activity of the LaFe_0.4_Ni_0.6_O_3_/CeO_2_ composite prepared via the Pechini route (sample 3) strongly increases and becomes even higher than the activity of composites 4 and 5 and the LaFe_0.4_Ni_0.6_O_3_ perovskite (sample 1).

The data obtained indicate that the catalytic activity of LaFe_0.4_Ni_0.6_O_3_/CeO_2_ composites depends on the sample preparation details and testing conditions.

In the low-temperature CO oxidation, the catalytic activity correlates mainly with the amount of weakly bound surface oxygen species that were reduced with hydrogen at low temperatures (up to 300 °C), which depends on the specific surface area values, chemical composition, and microstructure of the samples.

In the high-temperature CH_4_ oxidation (600–900 °C), when a bulk oxygen species may be involved in the reaction, the reducibility of the bulk samples and the rate of oxygen heteroexchange may influence the catalytic activity of the oxides [11,36]. The data obtained are in accordance with this observation. Therefore, the highest H_2_ consumption in the first peak (at 300–400 °C) was detected for pure LaFe_0.4_Ni_0.6_O_3_, while for composites 4 and 5 it was lower (Table 4); this correlates with activity of the samples in methane oxidation (Figure 7a). At T > 750 °C, the highest H_2_ consumption up to 600 °C was observed for sample 3 with a stronger modification of phases and a higher expected density of vacancies and interphase boundaries, which increase the reducibility of the samples, the oxygen transfer from the bulk to the surface, and the rate of oxygen heteroexchange according to [11,36], which affect the samples’ activity. Therefore, for high-temperature applications in oxidative catalytic reactions, the one-pot prepared composite may be attractive. Furthermore, the as-prepared composite material may be very attractive for SOFC application due to its high mixed conductivity according to [3,18].

## 4. Conclusions

The catalytic properties of LaFe_0.4_Ni_0.6_O_3_/CeO_2_ two-phase composite materials in oxidative reactions strongly depend on the details of their preparation that influence their reducibility and reaction conditions.

In the low-temperature (<600 °C) oxidation process (CO oxidation), the composites are very attractive because they are more active than perovskite or fluorite phases at nearly the same specific surface areas of the samples. There is a correlation between the activity and content of weakly bound surface oxygen species. A higher activity was demonstrated by the composite prepared via the mechanical treatment of the precursors of the perovskite and fluorite phases.

In the middle-temperature (600–750 °C) methane oxidation, perovskite is more attractive due to higher activity that correlates with its higher reducibility up to 400 °C.

In the high-temperature (>750 °C) methane oxidation, the one-pot prepared composite becomes attractive, in spite of its lower specific surface area, probably due to a higher content of vacancy in the bulk and interphase boundaries increasing the reducibility of the sample up to 600 °C.

## Figures and Tables

**Table 1:** Samples preparation conditions.

No.	Reagents for Samples Preparation	La/Ce	Preparation Details	Phase Composition
1	La, Fe, and Ni nitrate salts taken in a stoichiometric ratio	1/0	Prepared via the Pechini route precursor 1: milled and then calcined at 900 °C, 8 h	LaFe_0.4_Ni_0.6_O_3_
2	Ce nitrate salt	0/1	Prepared via Pechini route precursor 2: milled and then calcined at 900 °C, 8 h	CeO_2_
3	La, Fe, Ni, and Ce nitrate salts taken in a stoichiometric ratio	1/1	Prepared via one pot Pechini route precursor 3: milled and then calcined at 900 °C, 8 h	* LaFe_0.4_Ni_0.6_O_3_, * CeO_2_
4	Sample 1 + sample 2	1/1	Sample 1 and sample 2 with a La:Ce ratio = 1:1 were milled and then calcined at 900 °C, 8 h	LaFe_0.4_Ni_0.6_O_3_, CeO_2_
5	Precursor 1 + precursor 2	1/1	Precursor 1 and precursor 2 with a La:Ce ratio = 1:1 were milled and then calcined at 900 °C, 8 h	LaFe_0.4_Ni_0.6_O_3_, CeO_2_

* Bulk modified phases.

**Table 2:** Cell parameters and X-ray crystallite sizes for perovskite and fluorite phases in samples 1–5 (according to Table 1); specific surface area values (S_sp_, m^2^g^−1^) of the samples.

No	Cell Parameters and X-ray Crystallite Size for Phases						S sp., m^2^g^−1^
	LaFe_0.4_Ni_0.6_O_3_				CeO_2_		
	a, Å	b, Å	c, Å	CS, Å	a, Å	CS, Å	
1	5.507(1)	5.507(1)	13.304(2)	500			5.5
2					5.419(1)	450	6.8
3	5.544(3)	7.803(5)	5.453(3)	250			2.0
4	5.513(2)	5.513(2)	13.308(5)	400	5.423(1)	400	5.2
5	5.511(2)	5.511(2)	13.299(4)	400	5.419(1)	400	7.3

**Table 3:** Sample surface composition (at %), ratio of atomic concentrations of elements and ratio of oxygen states according to XPS data.

N	Sample	La/Ce	(Fe + Ni)/(Ce + La)	C, %	O, %	Fe, %	La, %	Ce, %	Ni, %	O_(529)_/O_(531)_
1	LaFe_0.4_Ni_0.6_O_3_	-	0.48	59.7	33.2	0.7	4.8	0	1.6	0.98
3	LaFe_0.4_Ni_0.6_O_3_/CeO_2_	1.06	0.24	40.5	45.4	1.2	5.8	5.5	1.6	2.07
4	LaFe_0.4_Ni_0.6_O_3_/CeO_2_	1.36	0.35	41.0	44.4	1.5	6.2	4.6	2.3	1.92
5	LaFe_0.4_Ni_0.6_O_3_/CeO_2_	0.83	0.29	40.4	45.2	1.1	5.1	6.1	2.1	2.47

**Table 4:** H_2_-TPR data.

Sample	Total Consumption Up to 900 °C, mol H_2_ g^−1^	Consumption in the First Peak, mol H_2_ g^−1^	First Peak Temperature Range, °C
1	6.14 × 10^−3^	1.25 × 10^−3^	60–400
2	1.17 × 10^−3^	0.15 × 10^−3^	60–550
3	2.81 × 10^−3^	1.38 × 10^−3^	60–600
4	3.14 × 10^−3^	0.64 × 10^−3^	60–400
5	3.17 × 10^−3^	0.65 × 10^−3^	60–400

## References

[1] Falcon H., Carbonio R.E., Fierro J.L.G. (2001). Correlation of Oxidation States in LaFe_x_Ni_1−x_O_3+δ_ oxides with catalytic activity for H_2_O_2_ decomposition. Catalysis.

[2] Konysheva E., Francis S.M., Irvine J.T.S. (2010). Crystal structure, oxygen nonstoichiometry, and conductivity of mixed ionic-electronic conducting perovskite composites with CeO_2_. J. Electrochem. Soc..

[3] Konysheva E., Irvine J.T.S. (2011). The La_0.95_Ni_0.6_Fe_0.4_O_3_-CeO_2_ system: Phase equilibria, crystal structure of components and transport properties. J. Solid State Chem..

[4] Bork A.H., Carrillo A.J., Hood Z.D., Yildiz B., Rupp J.L.M. (2020). Oxygen Exchange in Dual-Phase La_0.65_Sr_0.35_MnO_3_–CeO_2_ Composites for Solar Thermochemical Fuel Production. ACS Appl. Mater. Interfaces.

[5] Li X., Gao H. (2018). Role of ceria in the improvement of NO removal of lanthanum-based perovskite-type catalysts. RSC Adv..

[6] Kirchnerova J., Alifanti M., Delmon B. (2002). Evidence of phase cooperation in the LaCoO_3_–CeO_2_–Co_3_O_4_ catalytic system in relation to activity in methane combustion. Appl. Catal. A Gen..

[7] Forni L., Oliva C., Vatti F.P., Kandala M.A., Ezerets A.M., Vishniakov A.V. (1996). La-Ce-Co perovskites as catalysts for exhaust gas depollution. Appl. Catal. B Environ..

[8] Alifanti M., Blangenois N., Florea M., Delmon B. (2005). Supported Co-based perovskites as catalysts for total oxidation of methane. Appl. Catal. A Gen..

[9] Gellings P.J., Bouwmeester H.J.M. (1992). Ion and mixed conducting oxides as catalysts. Catal. Today.

[10] Stoian M., Rogé V., Lazar L., Maurer T., Védrine J.C., Marcu I.-C., Fechete I. (2021). Total Oxidation of Methane on Oxide and Mixed Oxide Ceria-Containing Catalysts. Catalysts.

[11] Pinaeva L.G., Isupova L.A., Prosvirin I.P., Sadovskaya E.M., Danilova I.G., Ivanov D.V., Gerasimov E.Y. (2013). La–Fe–O/CeO_2_ Based Composites as the Catalysts for High Temperature N_2_O Decomposition and CH_4_ Combustion. Catal. Lett..

[12] Provendier H., Petit C., Estoumes C., Kiennemann A. (1998). Dry reforming of methane. Interest of La-Ni-Fe solid solutions compared to LaNiO_3_ and LaFeO_3_. Stud. Surf. Sci. Catal..

[13] Provendier H., Petit C., Kiennemann A. (2001). Steam reforming of methane on LaNi_1-x_Fe_x_O_3_ (0 ≤ x ≤ 1) perovskites. Reactivity and characterisation after test. C. R. Acad. Sci. Paris Ser. IIc Chem./Chem..

[14] Kumar R., Coudhary R.J., Khan M.W., Srivastava J.P., Bao C.W., Tsai H.M., Chiou J.W., Asokan K., Pong W.F. (2005). Structural, electrical transport and x-ray absorption spectroscopy studies of LaFe_1−_*_x_*Ni_x_O_3_. J. Appl. Phys..

[15] Sukpirom N., Iamsaard S., Charojrochkul S., Yeyongchaiwat J. (2011). Synthesis and properties of LaNi_1-x_Fe_x_O_3-δ_ as cathode materials in SOFC. J. Mater. Sci..

[16] Bevilacqua M., Montini T., Tavagnacco C., Fonda E., Fornasiero P., Graziani M. (2007). Preparation, Characterization, and electrochemical properties of pure and composite LaNi_0.6_F_0.4_O_3_-based cathodes for IT-SOFC. Chem. Mater..

[17] Chiba R., Komatsu T., Orui H., Taguchi H., Nozawa K., Arai H. (2008). An SOFC cathode composed of LaNi_0.6_F_0.4_O_3_ and Ce(Ln)O_2_ (Ln = Sm, Gd, Pr). J. Korean Ceram. Soc..

[18] Yaroslavtsev I.Y., Bogdanovich N.M., Vdovin G.K., Dem’yanenko T.A., Bronin D.I., Isupova L.A. (2014). Cathodes based on rare-earth metal nickelate ferrites prepared from industrial raw materials for solid oxide fuel cells. Russ. J. Electrochem..

[19] Pavlova S., Kharlamova T., Sadykov V., Krieger T., Muzykantov V., Bespalko Y., Ishenko A., Rogov V., Belyaev V., Okhlupin Y. (2013). Structural features and transport properties of La(Sr)Fe_1-x_Ni_x_O_3-δ_–Ce_0.9_Gd_0.1_O_2-δ_ nanocomposites-advanced materials for IT SOFC cathodes. Heat Transf. Eng..

[20] Isupova L.A., Tsybulya S.V., Kryukova G.N., Alikina G.M., Boldyreva N.N., Yakovleva I.S., Ivanov V.P., Sadykov V.A. (2001). Real structure and catalytic activity of La_1-x_Ca_x_MnO_3+δ_ perovskites. J. Solid State Ion..

[21] Dulian P., Bąk W., Wieczorek-Ciurowa K., Kajtoch C. (2013). Controlled mechanochemical synthesis and properties of a selected perovskite-type electroceramics. Mater. Sci..

[22] Stojanovic B.D. (2003). Mechanochemical synthesis of ceramic powders with perovskite structure. J. Mater. Process. Technol..

[23] Zyryanov V.V., Sadykov V.A., Uvarov N.F., Alikina G.M., Lukashevich A.I., Neofitides S.G., Criado J.M. (2005). Mechanosynthesis of complex oxides with fluorite and perovskite-related structures and their sintering into nanocomposites with mixed ionic–electronic conductivity. Solid State Ion..

[24] Isupova L.A., Obyskalova E.A., Rogov V.A., Tsybulya S.V., Dovlitova L.S., Burgina E.B., Ischenko A.V., Zaikovskii V.I., Sadykov V.A., Orlovskaya N. (2006). Doped ceria—LaMeO_3_ (Me = Mn, Fe, Co) nanocomposites: Synthesis via mechanochemical activation route and properties. Mater. Res. Soc. Symp. Proc..

[25] Gorbunova V.A., Sliapniova L.M., Gorbunov A.V. (2020). Thermochemical Preparation and Properties of Low-Cost Polylanthanide Manganite Materials of Ln(La, Ce, Nd, Pr)_x_Ca_y_MnO_3_-Type with Perovskite-Fluorite Structure. Sci. Tech..

[26] Zhao Z., Zou M., Huang H., Wofford H., Tong J. (2021). Stable perovskite-fluorite dual-phase composites synthesized by one-pot solid-state reactive sintering for protonic ceramic fuel cells. Ceram. Int..

[27] Ohzeki T., Hashimoto T., Shozugawa K., Matsuo M. (2010). Preparation of LaNi_1-x_Fe_x_O_3_ Single Phase and Characterization of their Phase Transition Behaviors. Solid State Ion..

[28] Pechini M.P. (1967). Method of Preparing Lead and Alkaline Earth Titanates and Niobates and Coating Method Using the Same to Form a Capacitor. US Patent.

[29] Tsybulya S.V., Cherepanova S.V., Solovʹeva L.P. (1996). Polycrystal Software Package for IBM/PC. J. Struct. Chem..

[30] Scofield J.H. (1976). Hartree-Slater subshell photoionization cross-sections at 1254 and 1487 eV. J. Electron Spectrosc. Relat. Phenom..

[31] XPSPEAK 4.1. http://xpspeak.software.informer.com/4.1/.

[32] Ren H., Wang Z., Chen X., Jing Z., Qu Z., Huang L. (2021). Effective mineralization of p-nitrophenol by catalytic ozonation using Ce-substituted La_1−x_Ce_x_FeO_3_ catalyst. Chemosphere.

[33] Isaacs M.A., Davies-Jones J., Davies P.R., Guan S., Lee R., Morgan D.J., Palgrave R. (2021). Advanced XPS characterization: XPS-based multi-technique analyses for comprehensive understanding of functional materials. Mater. Chem. Front..

[34] van der Heide P.A.W. (2002). Systematic x-ray photoelectron spectroscopic study of La_1-x_Sr_x_-based perovskite-type oxides. Surf. Interface Anal..

[35] Yakovleva I.S., Isupova L.A., Rogov V.A., Sadykov V.A. (2008). Forms of oxygen in La_1−x_Ca_x_MnO_3+δ_ (x = 0–1) perovskites and their reactivities in oxidation reactions. Kinet. Catal..

[36] Liu W., Flytzanistephanopoulos M. (1995). Total Oxidation of Carbon-Monoxide and Methane over Transition Metal Fluorite Oxide Composite Catalysts. J. Catal..

[37] Ivanov D.V., Pinaeva L.G., Sadovskaya E.M., Isupova L.A. (2011). Influence of the mobility of oxygen on the reactivity of La_1−*x*_Sr*_x_*MnO_3_ perovskites in methane oxidation. Kinet. Catal..

